# Anatomical insights beyond the centre edge angle in borderline hip dysplasia: A computerised tomography study

**DOI:** 10.1002/jeo2.70268

**Published:** 2025-05-19

**Authors:** Joaquín Lara, Alejandro Neira, Alan Garín, Javier del Río, Alexander Tomic, Nicolás García, Matías Roby, Carlos De la Fuente

**Affiliations:** ^1^ Hip Center, Clinica Las Condes Santiago Chile; ^2^ Escuela de Kinesiologia, Facultad de Medicina y Ciencias de la Salud, Universidad Mayor Santiago Chile; ^3^ Orthopeadics, Innovation Center, Clínica MEDS Santiago Chile; ^4^ Radiology, Clínica MEDS Santiago Chile; ^5^ Exercise and Rehabilitation Sciences Institute, Postgraduate, Faculty of Rehabilitation Sciences Universidad Andres Bello Santiago Chile

**Keywords:** anatomy, biomechanics, hip, images, surgery

## Abstract

**Purpose:**

Borderline hip dysplasia (BhD) may be associated with insufficient acetabular coverage. Thus, we investigated potential differences in acetabular anatomical measurements derived from computerised tomography (CT) that characterise BhD compared with healthy controls.

**Methods:**

BhD patients (lateral centre edge angle [LCEA] between 18° and 25°) and healthy controls (LCEA between 25° and 40°) underwent anteroposterior pelvic X‐ray and CT scans to study the Wiberg and Tönnis angle, the extrusion and Fear indices, notch width and depth, anterior and posterior wall heights, anterior and posterior articular surfaces, articular circumference, the ratio between the anterior articular surface and the articular circumference, the ratio between the posterior articular surface and the articular circumference, and the ratio between the notch width and the articular circumference. Independent two‐tailed *t*‐tests, Mann–Whitney *U* tests, and odds ratios were obtained (*α* = 5%).

**Results:**

Twenty‐three BhD patients (aged 31.5 ± 8.3 years and LCEA 21.6° ± 4.0°) and thirty‐one healthy controls (aged 34.1 ± 8.0 years and LCEA 33.7° ± 5.5°) were included. The CT features most sensitive for detecting BhD were the anterior acetabular surface (*p* < 0.001) and ratios of anterior (*p* = 0.009), anteroposterior (*p* = 0.008) and posterior (*p* < 0.008) acetabular surfaces, as well as acetabular notch width (*p* = 0.002).

**Conclusions:**

CT characterisation showed insufficient acetabular coverage in BhD patients along the superior axis (lower Wiberg angle, and increased Tönnis angle and extrusion index), anterior axis (lower anterior acetabular surface and anteroposterior acetabular surface ratio, and increased posterior acetabular ratio), and inferior axis (increased acetabular notch width). These structural alterations suggest that periacetabular osteotomy may address persistent pathological deformation and stress on soft tissues and cartilage‐bone structures more effectively.

**Level of Evidence:**

Diagnostic III.

**Abbreviations**
AP: antero‐posterior
AP: antero‐posterior
BhD: borderline hip dysplasia
CI: 95% of confidence interval for mean differences between groups
CI: 95% of confidence interval for mean differences between groups
CT: computerised tomography
CT: computerised tomography
DICOM: digital imaging and communications in medicine
ICC: intraclass correlation coefficient
LCEA: lateral centre edge angle
mm: millimetre
mm: millimetre
β: statistical error type II
α: statistical error type I
°: sexagesimal degree
∆: mean difference between groups
%: percent

## INTRODUCTION

Borderline hip dysplasia (BhD) is defined as a lateral centre edge angle (LCEA) of 18°–25°, which reflects lateral borderline acetabular coverage [8, 22]. It can affect 19.8%–23.3% of the asymptomatic general population and 12.8% of symptomatic patients [10]. Diminished acetabular coverage constitutes a complex musculoskeletal developmental defect that leads to pathological forces, increased stress concentration [10, 23], labral tearing, cartilage attrition [10], and a higher risk of early end‐stage osteoarthritis [3, 5].

BhD patterns [8] include lateral and posterolateral acetabular under coverage, and normal lateral acetabular coverage [8, 11]. An anterior coverage deficit is another frequent finding [8, 16, 25] and has been associated with poorer joint restoration following periacetabular osteotomy [15]. A previous report suggested that BhD may be well characterised by anterior femoral coverage [13]. However, the LCEA or Wiberg angle [8, 23] alone does not provide information on non‐lateral acetabular coverage alterations [5]. LCEA is the standard radiographic measure used to assess the lack of lateral femoral coverage, which is the most common finding in severe hip dysplasia [5, 8]. Consequently, BhD may mischaracterized due to an incomplete representation of its tridimensional coverage architecture, overshadowing morphology alterations [8, 16]. This structural underrepresentation could contribute to inconsistent and variable treatment decisions (e.g., inappropriate selection of periacetabular osteotomy or insufficient arthroscopy procedures) [5], ultimately affecting patient outcomes.

Deficits in femoral coverage morphology in BhD remain a topic of ongoing discussion [1]. Additional morphological descriptors related to anteroposterior coverage, such as notch, wall height, articular surfaces and circumference, the ratios between articular surfaces and circumference, and the ratio between the notch width and the articular circumference, may provide a better characterisation of BhD patients. Consequently, computerised tomography (CT) might help explore the acetabular architecture in BhD beyond the LCEA [14, 20]. Therefore, we aim to investigate potential differences in acetabular anatomical measurements derived from CT images that characterise BhD compared with healthy controls. We hypothesise that notch, wall height, articular surfaces and circumference, the ratios between articular surfaces and circumference, and the ratio between the notch width and the articular circumference would characterise better BhD compared to healthy controls.

## MATERIALS AND METHODS

### Study design and setting

In this case‐control, cross‐sectional study, we prospectively recruited participants from the same institution between 2018 and 2023, following *a priori* sample size estimations. We included two independent groups, aged between 18 and 40 years, and recruited in parallel. The BhD group was defined as having an LCEA between 18° and 25°, and the healthy group as having an LCEA between 25° and 40°. All participants underwent anteroposterior pelvic X‐ray and CT imaging. Notch width and depth, wall height, articular surfaces and circumference, the ratios between articular surfaces and circumference, and the ratio between the notch width and the articular circumference were compared between groups.

Finally, this study adhered to the ethical principles of the Declaration of Helsinki regarding human experimentation, was approved by the institutional ethical committee of MEDS clinic, and written informed consent was obtained from all participants. Additionally, the study was based on the STROBE statement for original research.

### Participants

This study included patients with clinical records of BhD treated by the same orthopaedic team, aged between 18 and 40 years, who were capable of walking upstairs and downstairs without assistance, had a positive test for anterior hip pain, an abnormal foot progression angle walking, and available anteroposterior pelvic X‐ray records. Age‐matched healthy participants were included if they did not have a dysplasia diagnosis, were between 18 and 40 years old (consistent with previous reports [24]), and had the capacity to walk upstairs and downstairs without assistance. The exclusion criteria for both groups included any history of the spine, pelvis, or lower limb fractures, chronic musculoskeletal disorders, soft tissue alterations, and any cardiorespiratory or neurologic pathology. The exclusion criteria for patients were any orthopaedic conditions other than dysplasia, leg length difference greater than 1.5 cm, and failure to habitually use orthopaedic shoes to correct the length difference. The exclusion criteria for the healthy group were a positive orthopaedic test for anterior hip pain, an abnormal foot progression angle during walking, and any radiological sign of osteoarthritis.

A priori estimated sample size was made for a two‐tailed independent t‐test comparing two groups, a large effect size (0.857) obtained from the mean values of the anterior uncovered (0.25) and covered (0.46) index, based on an estimated mean dispersion of 0.245 previously described [8], with an alpha level of 5% and a statistical power of 80%. The minimum total estimated sample was 46 participants (23 per group), corresponding to a critical *t*‐value of 2.02. The estimation was made using the G*Power software version 3.1.9.2 (Universität Kiel, Germany).

### Procedures

One of four orthopaedic surgeons specialising in hip preservation clinically evaluated the patients, and their previous records and images of dysplasia were also checked. At this time point, sociodemographic data (age, weight and sex) were collected, and new anteroposterior pelvis X‐ray and CT images were requested (LCEA, Wiberg and Tönnis angle, and Extrusion and fear index were obtained). Orthopaedic surgeons confirmed the BhD diagnosis in those who obtained an LCEA < 25° who fulfilled all inclusion and exclusion criteria. Healthy participants from the community were invited to participate. Both patients and healthy participants underwent a CT study using a 16‐slice CT (Brivo CT 385 series, G.E. Healthcare, USA) with a resolution of 0.62 mm and pixel spacing of 0.43 mm × 0.43 mm.

A blinded senior musculoskeletal radiologist and orthopaedic surgeon manually measured ten specific acetabulum features derived from axial CT scans (mean value of three measurements). The measurements were taken in the horizontal plane through the centre of the femoral head. The centre of the femoral head was determined through visual exploration using linked and synchronised horizontal‐coronal views to ensure the highest femoral diameter. All image data were exported in DICOM format and analysed using Philips Radiology Information System software version 11 (Philips Medical Systems, Netherlands).

### Outcomes

**Figure 1:**
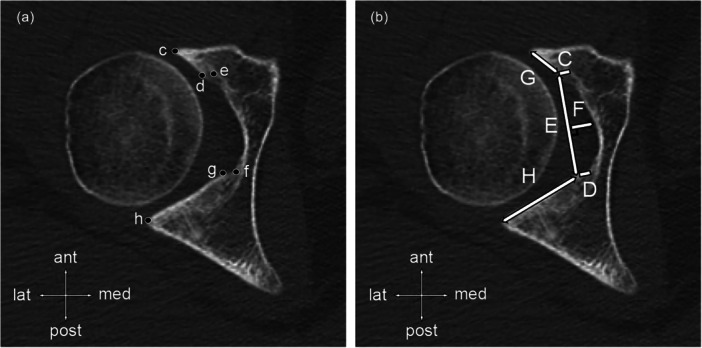
Computed tomography (CT) landmarks and measurements are in reference to the axial hip plane. (a) CT landmarks are shown in c as the anterior border of the anterior lunate surface, d as the posterior border of the anterior lunate surface, e as the anterior edge of the acetabular fossa, f as the posterior edge of the acetabular fossa, g as the anterior border of posterior lunate surface, and has the posterior border of the posterior lunate surface. (b) CT distances measured in this study are shown in C as anterior lunate wall height, D as posterior lunate wall height, E acetabular notch width, F acetabular fossa depth, G as anterior lunate surface, and H as posterior lunate surface.

The anatomical measurements are summarised in Figure 1 and defined as follow: (1) acetabular notch width defined by the distance between the posterior border of anterior lunate surface and the anterior border of posterior lunate surface measured in millimetres, (2) acetabular fossa depth defined as the distance drawn from the midpoint of the acetabular notch width line with a perpendicular line to the bottom of the notch measured in millimetres, (3) anterior lunate wall height defined as the distance from the anterior edge of acetabular fossa to the posterior border of anterior lunate surface measured in millimetres, (4) posterior lunate wall height defined as the distance from the posterior edge of acetabular fossa to the anterior border of posterior lunate surface measured in millimetres, (5) anterior lunate surface width defined as the distance between anterior and posterior borders of anterior lunate surface measured in millimetres, (6) posterior lunate surface defined as the distance between anterior and posterior borders of posterior lunate surface measured in millimetres, (7) total lunate surface defined as the sum of anterior and posterior lunate surfaces, (8) anteroposterior lunate surface ratio defined as the ratio of the anterior and posterior lunate surfaces, (9) lunate anterior/total surface ratio defined as the ratio of the anterior and total lunate surfaces and (10) lunate posterior/total surface ratio defined as the ratio of the anterior and total lunate surfaces.

### Statistical analysis

Normality and homoscedasticity principles were checked through Shapiro–Wilk and Levene's tests. Consequently, data were described as the mean and standard deviation for normally distributed variables with homogeneity. Variables that were not normally distributed or did not meet the homoscedasticity assumption were described using the median and interquartile range.

Group comparisons were conducted using an independent two‐tailed *t*‐test for normally distributed variables with homogeneity, while Mann–Whitney *U* test was used for variables that were not normally distributed or lacked homogeneity. Effect sizes (Hedges' g for different sample sizes) and confidence intervals were described. Effect size interpretation followed standardised thresholds: small (0.2–0.5), moderate (0.5–0.8) and large ( > 0.8).

Odds ratios were calculated for statistically significant acetabular anatomical features relative to the Wiberg angle. The effect size was estimated as the ln(odds)/1.81 [6] with confidence intervals for odds ratios, and *p*‐values determined using the one‐tailed Fisher exact test were also estimated.

We assessed the intraclass correlation coefficient (ICC) and the 95% confidence intervals to determine the reliability of the intra‐ and inter‐observer CT measurements. Measurements were performed at two separate time points at least two weeks apart, with blinded clinical information, in an independent sample of 10 participants (*α* = 5% and *β* = 20%) [4]. All statistical significance levels were set at *α* = 5%. All statistical analyses were performed using SPSS 20.0 software (I.B.M. Inc., U.S.A.).

## RESULTS

Fifty‐four participants were included in this study. Twenty‐three patients and 31 participants were included in the BhD and healthy control groups, respectively. Eight participants in the BhD group withdrew after undergoing CT imaging. The BhD group consisted of three men and twenty women aged 31.5 ± 8.3 years and LCEA 21.6° ± 4.0°. The healthy control group consisted of ten men and twenty‐one women aged 34.1 ± 8.0 years and LCEA 33.7° ± 5.5°. Radiological measurements for both groups are described in Table 1.

**Table 1:** Radiological and computerised tomography (CT) outputs of the study.

	Borderline dysplasia	Healthy control	∆	95%CI	Effect size
**Radiological measurements**					
Wiberg angle,°	23.0 [3]^b^	34.0 [6]	12.1	9.5–14.7	2.5
Tönnis angle,°	12.0 ± 4.4^b^	7.8 ± 2.7	4.2	2.3–6.1	1.2
Extrusion Index, %	68.0 [13]^b^	83.0 [8]	19.5	12.3–26.7	1.7
Fear Index,°	6 [6]	10.0 [8]	0.7	−5.0 to 6.3	0.1
**CT measurements**					
Acetabular notch width, mm	31.0 [6.1]^a^	27.7 [3]	3.2	1.0–5.1	0.9
Acetabular fossa depth, mm	9.3 [2.6]	8.3 [1.5]	0.4	−1.0 to 0.4	0.3
Anterior lunate wall height, mm	3.4 ± 0.7	3.4 ± 1.3	0.03	−0.51 to 0.62	0.03*
Posterior lunate wall height, mm	4.4 [2.3]	4.2 [1.4]	0.8	−0.3 to 2.0	0.4
Anterior lunate surface, mm	12.1 ± 2.1^b^	15.0 ± 3.0	2.9	1.4–4.4	1.1
Posterior lunate surface, mm	21.8 ± 3.3	23.5 ± 3.6	1.8	−0.2 to 3.7	0.5
AP lunate surface ratio, mm/mm	0.56 ± 0.1^a^	0.64 ± 0.1	0.08	0.02–0.14	0.8
Lunate Anterior/total surface ratio, mm/mm	0.35 ± 0.04^a^	0.39 ± 0.05	0.03	0.01–0.06	0.8
Lunate posterior/total surface ratio, mm/mm	0.64 ± 0.04^a^	0.61 ± 0.05	0.02	0.01–0.06	0.8
Total lunate surface, mm	39.1 ± 4.2	38.5 ± 5.7	1.4	−3.3 to 2.1	0.1

*Note*: Data are described as mean ± standard deviation and median [interquartil range].

Abbreviations: 95%CI, 95% of confident interval for mean differences between groups; AP, antero‐posterior; CT, computerised tomography; mm, millimetre; **∆**, mean difference between groups; °, sexagesimal degree; %, percent.

^a^ Statistical significance for Displasia Group compared with Controls (*p* < 0.05).

^b^ Statistical significance for Displasia Group compared with Controls (*p* < 0.001).

* Two decimal places were shown to highlight the magnitude of the effect size.

**Figure 2:**
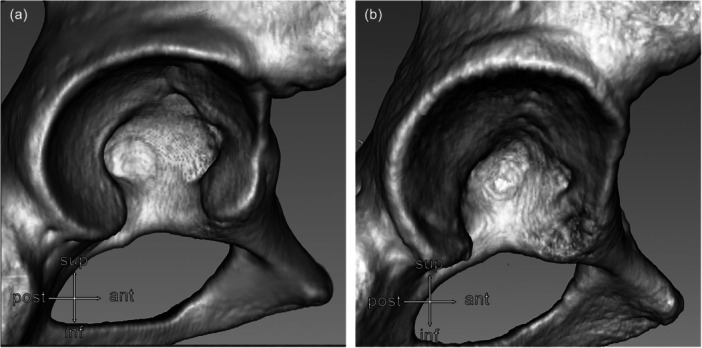
Three‐dimensional CT image renderization of the acetabulum. The images illustrate the anatomical differences observed in the anterior lunate surface and acetabular notch width with a preserved posterior lunate surface between a healthy subject (a) and a BhD patient (b). BhD, borderline hip dysplasia; CT, computerised tomography.

The acetabular CT images are shown in Figure 2. The BhD group had a lower Wiberg angle (*p* < 0.001; large effect size), anterior acetabular surface (*p* < 0.001; large effect size), anterior (*p* = 0.009; large effect size) and anteroposterior (*p* = 0.008; large effect size) acetabular surface ratio compared with healthy controls (Table 1). The BhD group exhibited a higher Tönnis angle (*p* < 0.001; large effect size), extrusion index (*p* < 0.001; large effect size), acetabular notch width (*p* = 0.002; large effect size), and posterior acetabular surface ratio (*p* < 0.008; large effect size) compared with healthy controls (Table 1).

The odds ratio for acetabular notch width was 2.1 [95%CI: 0.69–6.66] with *p* = 0.147 and an effect size of 0.41 (small effect size). For the anterior acetabular surface, the odds ratio was 11.2 [95%CI: 2.23–56.17] with *p* = 0.001 and an effect size of 1.33 (large effect size). For the anteroposterior acetabular surface ratio, the odds ratio was 2.8 [95%CI: 0.83–8.54] with *p* = 0.083 and an effect size of 0.57 (moderate effect size). For the anterior acetabular surface ratio, the odds ratio was 3.0 [95%CI: 0.94–9.71] with *p* = 0.053 and an effect size of 0.61 (moderate effect size). For the posterior acetabular surface ratio, the odds ratio was 3.0 [95%CI: 0.94–9.71] with *p* = 0.053 and an effect size of 0.61 (moderate effect size). The intra‐ and inter‐observer reliability measurements are described in Table 2.

**Table 2:** Reliability measures of the outcomes.

	Intraobserver		Interobserver	
	Borderline dysplasia	Healthy control	Borderline dysplasia	Healthy control
	ICC (95% CI)	ICC (95% CI)	ICC (95% CI)	ICC (95% CI)
**CT measurements**				
Acetabular notch Width, mm	0.36 (0.70–0.81)	0.98 (0.93–0.99)	0.53 (0.36–0.87)	0.71 (0.01–0.92)
Acetabular fossa depth, mm	0.83 (0.34–0.95)	0.93 (0.75–0.98)	0.78 (0.22–0.94)	0.80 (0.24–0.95)
Anterior lunate wall height, mm	0.83 (0.26–0.95)	0.92 (0.71–0.98)	0.77 (0.17–0.94)	0.89 (0.39–0.97)
Posterior lunate wall height, mm	0.82 (0.35–0.95)	0.96 (0.86–0.99)	0.79 (0.24–0.94)	0.93 (0.74–0.98)
Anterior lunate surface, mm	0.93 (0.73–0.98)	0.98 (0.92–0.99)	0.83 (0.38–0.95)	0.92 (0.73–0.98)
Posterior lunate surface, mm	0.94 (0.77–0.98)	0.98 (0.91–0.99)	0.89 (0.56–0.74)	0.96 (0.87–0.99)
AP anterioposterior lunate surface ratio, mm/mm	0.96 (0.86–0.92)	0.98 (0.94–0.99)	0.91 (0.62–0.98)	0.94 (0.78–0.98)
Lunate anterior/total surface ratio, mm/mm	0.95 (0.80–0.98)	0.98 (0.94–0.99)	0.88 (0.48–0.97)	0.94 (0.78–0.98)
Lunate posterior/total surface ratio, mm/mm	0.95 (0.80‐0.98)	0.98 (0.93‐0.96)	0.88 (0.48‐0.97)	0.95 (0.88–0.98)
Total lunate surface, mm	0.93 (0.72‐0.98)	0.99 (0.96‐0.99)	0.84 (0.4‐0.96)	0.96 (0.87–0.99)

Abbreviations: AP, antero‐posterior; CI, confident interval; ICC, intraclass correlation coefficient; mm, millimetre; %, percent.

## DISCUSSION

The main findings of our study were that BhD patients develop pathological characteristics associated with diminished: (1) anterior acetabular coverage (lower anterior acetabular surface and anteroposterior acetabular surface ratio, and increased posterior acetabular ratio), (2) lateral acetabular coverage (lower Wiberg angle, and increased Tönnis angle and extrusion index), (3) inferior acetabular coverage (increased acetabular notch width), (4) BhD patients are 2.8–11.2 times more likely to develop diminished anterior acetabular coverage and (5) BhD patients are 2.1 times more likely to develop diminished inferior acetabular coverage. These anatomical changes may compromise anterior, superior and inferior joint stability, leading to pathological acetabular stress distribution [23] and non‐physiological femoral head migration within the joint. This pattern resembles micro‐instability or severe osteoarthritis, as previously described in the literature [9, 12, 19, 21]. From a Clinical implication perspective, acetabular reorientation techniques, such as periacetabular osteotomy, should primarily focus on three‐dimensional reorientation and coverage augmentation to improve joint stability and normalise stress distribution [7]. This approach may be more effective in preserving the acetabulum than prioritising non‐structural repairs or reconstructions of secondary stabilisers, where pathological forces induced by altered orientations persist.

The lower anterior acetabular surface and anteroposterior acetabular surface ratio, and increased posterior acetabular ratio were sensitive indicators of diminished anterior acetabular coverage in our study. These findings align with anatomical observations in female dysplasia [16, 17]. The anterior acetabular surface was the most sensitive variable, with a ratio of 11.2 times, indicating a high risk (21/23 [91.3%]) of diminished anterior acetabular coverage in BhD patients compared to healthy controls. Surgical planning may include reorientation procedures, such as periacetabular osteotomy or reverse periacetabular osteotomy in cases of excessive anteversion, to reposition the acetabulum while preserving the hip joint. Additionally, acetabuloplasty, employing an osteochondral graft to augment the deficient acetabular rim, could enhance coverage and load distribution. Adjunct procedures following reorientation, including labral repair, ligament reconstruction and capsular tightening, may further enhance joint stability and load distribution.

The greater acetabular notch width observed in BhD patients compared to healthy controls suggests that incomplete anterior acetabular coverage influences the appropriate inferior coverage, that is, by a lack of anterior acetabular horn (Figure 1b), increasing the notch width. The ratio of developing increased acetabular notch width is 2.1 times higher, with an increased risk of 16/23 [69.6%] in BhD patients compared to healthy controls. The acetabular notch and the transverse acetabular ligament are physiological barriers against inferior dislocation [18]. The transverse acetabular ligament and the labrum partially support the inferior load‐bearing during femoral head movements [2]. Therefore, an increased acetabular notch width may represent a new contributing factor to micro‐instability or hypermobility [9, 12, 19, 21]. This issue may be addressed using combined surgical strategies, such as acetabular notch and horn plasty for similar cases as Figure 1b illustrates, labral reconstruction, and augmentation of the transverse ligament, teres ligament, and pubofemoral ligament to enhance inferior stability. This combined approach remains an emerging strategy that warrants further clinical investigation. To our knowledge, inferior micro‐instability in BhD patients has not been previously discussed.

Nowadays, better improved and personalised anatomical patient study might orientate the reorientation of acetabular surgery to restore hip stability and hip joint preservation. These studies include biomechanical simulations for stress distribution restoration, tridimensional visualisation, printing, and artificial intelligence to support pre‐surgical planning and management. Another future effort is to find algorithms that allow the projection of sensitive CT features to traditional dysplasia radiology.

We know our study is not without limitations. The primary limitation is that our sample primarily consisted of female BhD patients, which may explain why the posterolateral lack of coverage, typically associated with males, was not observed. Additionally, CT scanning does not replicate the weight‐bearing conditions captured by X‐ray imaging, and pelvic positioning could influence coverage measurements. For instance, reduced anterior coverage may not be significant in the presence of an anteverted pelvis. Assessing pelvic rotation using the anterior pelvic plane and pelvic tilt on the sagittal plane would be beneficial. Future studies incorporating pelvic incidence and morphotype classification, such as the Bordeaux classification, could provide a more comprehensive understanding of hip coverage. Lastly, cadaveric and biomechanical simulations are needed to explore further the intensity and consequences of instability in BhD patients.

## CONCLUSION

The most sensitive CT‐based features of the acetabular surface were the anterior acetabular surface, the acetabular notch width, and the anterior, anteroposterior, and posterior acetabular surface ratios. CT characterisation showed insufficient acetabular coverage can be found along the superior (lower Wiberg angle, and increased Tönnis angle and extrusion index), anterior (lower anterior acetabular surface and anteroposterior acetabular surface ratio, and increased posterior acetabular ratio), and inferior (increased acetabular notch width) axis in BhD patients. Clinical implications suggest that acetabular reorientation and coverage augmentation should primarily aim to normalise joint stability and stress distribution to prolong the preservation of the acetabulum. Adjunct procedures after reorientation, including labral repair, ligament reconstruction, and capsular tightening, may further enhance joint stability and overall function.

## AUTHOR CONTRIBUTIONS

*Conceptualisation*: Joaquín Lara, Javier del Río, Alexander Tomic, Alan Garín, Alejandro Neira, and Carlos De la Fuente. *Data curation*: Nicolás García, Alejandro Neira, and Carlos De la Fuente. *Formal analysis*: Alejandro Neira and Carlos De la Fuente. *Funding acquisition*: Alejandro Neira. *Investigation*: Joaquín Lara, Javier del Río, Alexander Tomic, Alan Garín, Alejandro Neira, and Carlos De la Fuente. *Methodology*: Alejandro Neira and Carlos De la Fuente. *Project administration*: Alan Garín, Matías Roby, Alejandro Neira, and Carlos De la Fuente. *Resources*: Alejandro Neira and Carlos De la Fuente. *Software*: Alejandro Neira and Carlos De la Fuente. *Supervision*: Carlos De la Fuente. *Validation*: Joaquín Lara, Javier del Río, Alexander Tomic, Alan Garín, Nicolás García, Matías Roby, Alejandro Neira, and Carlos De la Fuente. *Visualisation*: Alejandro Neira and Carlos De la Fuente. *Roles/writing–original draft*: Carlos De la Fuente. *Writing–review and editing*: Joaquín Lara, Javier del Río, Alexander Tomic, Alan Garín, Alejandro Neira, and Carlos De la Fuente.

## CONFLICT OF INTEREST STATEMENT

The authors declare no conflicts of interest.

## ETHICS STATEMENT

This study adhered to the ethical principles of the Declaration of Helsinki regarding human experimentation, was approved by the institutional ethical committee of MEDS clinic, and written informed consent was obtained from all participants.

## Data Availability

Original data sets will be available by Joaquín Lara.
